# Manipulative therapy in addition to usual medical care accelerates recovery of shoulder complaints at higher costs: economic outcomes of a randomized trial

**DOI:** 10.1186/1471-2474-11-200

**Published:** 2010-09-06

**Authors:** Gert JD Bergman, Jan C Winter, Maurits W van Tulder, Betty Meyboom-de Jong, Klaas Postema, Geert JMG van der Heijden

**Affiliations:** 1 Department of General Practice, University Medical Center Groningen, Groningen, The Netherlands; 2 Centre for Rehabilitation, University Medical Center Groningen, Groningen, The Netherlands; 3 Institute for Research in Extramural Medicine, VU Medical Centre Amsterdam, the Netherlands; 4 Julius Centre for Health Sciences and Primary Care, University Medical Centre Utrecht, the Netherlands

## Abstract

**Background:**

Shoulder complaints are common in primary care and have unfavourable long term prognosis. Our objective was to evaluate the clinical effectiveness of manipulative therapy of the cervicothoracic spine and the adjacent ribs in addition to usual medical care (UMC) by the general practitioner in the treatment of shoulder complaints.

**Methods:**

This economic evaluation was conducted alongside a randomized trial in primary care. Included were 150 patients with shoulder complaints and a dysfunction of the cervicothoracic spine and adjacent ribs. Patients were treated with UMC (NSAID's, corticosteroid injection or referral to physical therapy) and were allocated at random (yes/no) to manipulative therapy (manipulation and mobilization). Patient perceived recovery, severity of main complaint, shoulder pain, disability and general health were outcome measures. Data about direct and indirect costs were collected by means of a cost diary.

**Results:**

Manipulative therapy as add-on to UMC accelerated recovery on all outcome measures included. At 26 weeks after randomization, both groups reported similar recovery rates (41% vs. 38%), but the difference between groups in improvement of severity of the main complaint, shoulder pain and disability sustained. Compared to the UMC group the total costs were higher in the manipulative group (€1167 vs. €555). This is explained mainly by the costs of the manipulative therapy itself and the higher costs due sick leave from work. The cost effectiveness ratio showed that additional manipulative treatment is more costly but also more effective than UMC alone. The cost-effectiveness acceptability curve shows that a 50%-probability of recovery with AMT within 6 months after initiation of treatment is achieved at €2876.

**Conclusion:**

Manipulative therapy in addition to UMC accelerates recovery and is more effective than UMC alone on the long term, but is associated with higher costs.

**International Standard Randomized Controlled Trial Number Register:**

ISRCTN11216

## Background

Shoulder complaints are common. In a recent cross-sectional study 21% of the general population reported shoulder complaints [1]. Shoulder complaints are characterized by functional disability, most often due to shoulder pain or to restricted range of motion in the shoulder. This results in substantial consumption of medical health care, sick leave from work and disability in daily living [1,2]. In the Netherlands, general practitioners treat shoulder complaints according to the National Guidelines of the Dutch College of General Practitioners [3,4]. This treatment is beneficial on the short term, but cannot prevent an unfavourable long term prognosis: only 50% of all new episodes of shoulder complaints resolve within six months, while at 12 months still over 40% of all patients are disabled during work and leisure time [5,6].

Musculoskeletal disorders are the second most expensive group for health care costs in the Netherlands. Shoulder complaints constitute the third most largest group of musculoskeletal disorders [7]. Insurance data from Sweden indicate that 18% of disability payments made for musculoskeletal disorders are spent on shoulder complaints [8]. Therefore, there is a need for investigating the cost-of-illness of treatment of shoulder complaints. We conducted an economic evaluation alongside a randomized trial. The objective was to evaluate the clinical effectiveness of manipulative therapy of the cervicothoracic spine and the adjacent ribs in addition to usual medical care (UMC) by the general practitioner in the treatment of shoulder complaints compared to UMC alone. The design of the study and the clinical outcomes are reported elsewhere [9,10]. The study was approved by the Medical Ethical Committee of the University Medical Centre Groningen.

## Methods

### Participants and randomization

Potential eligible participants with shoulder complaints were recruited in 50 general practices in the province of Groningen, The Netherlands. At presentation the general practitioner checked the criteria for eligibility, informed the researcher (GJDB) of each eligible patient, and initiated treatment (UMC). The general practitioner used a standardized eligibility checklist and a physical examination as recommended by the practice guidelines for shoulder disorders issued by the Dutch College of General Practitioners [4,11]. A baseline assessment at the research centre was scheduled within two weeks of presentation. Shoulder complaints were defined as pain at rest or during movement of the upper arm in the area between the neck and the elbow. There was no limitation in the duration of complaints at first presentation, while radiating pain to the neck-region or to the lower part of the arm was no reason to exclude the patient. By pain or restricted movement during physical examination, presence of both shoulder complaints and dysfunction of the cervicothoracic spine and the adjacent ribs was confirmed. Eligible patients were 18 years of age or older, and had had no consultation or treatment for shoulder complaints in the past three months. Reasons for exclusion were: acute severe trauma such as fractures, ruptures or dislocation in the shoulder-region, previous (orthopaedic) surgery in the shoulder region, clear treatment preference deviating from study treatments, contraindications for manipulative therapy (e.g. hyper-mobility, instability or severe arthrosis of the cervicothoracic spine), signs of cervical nerve root compression, presence of specific rheumatic disorders, presence of dementia or other severe psychiatric, emotional or behavioural disorders, shoulder disorders due to general internal thoracic and abdominal pathology and inability to complete Dutch written questionnaires. In the research centre the conducting researcher (GJDB) verified the entry criteria before randomization by a structured medical history and a physical assessment. Patients were evenly allocated to either manipulative therapy additional to usual medical care (UMC) or to UMC alone after verification of eligibility and consent. Thereafter, the researcher (GJDB) opened pre-prepared numbered opaque sealed envelopes containing the treatment allocation code from a randomization list produced by independent physician.

### Interventions

All patients received *usual medical care *(*UMC) *from their general practitioner (GP). All participating GPs received training to apply UMC as encouraged by Guidelines for Shoulder Complaints of the Dutch College of General Practitioners [4]: during the first two weeks information regarding the nature and the course of shoulder complaints was given, together with advises how to use the affected shoulder in daily living, supplemented with analgesics or NSAID's if necessary. If limited effect ensued from this approach, up to 3 corticosteroid injections (in subacromial space or glenohumeral joint) could be given. Physiotherapy (9 treatment sessions in a 3-months period) was considered for complaints persisting for 6 weeks or more and consisted of treatment of the shoulder with exercises, massage and physical applications.

*Additional manipulative therapy *consisted of specific manipulation techniques (high velocity, low amplitude thrust) and mobilization techniques (passive low velocity movements within the range of motion of the joint) of the cervical spine, the upper thoracic spine and the upper ribs on the segmental level and was aimed to restore normal spinal function [12,13]. A maximum of 6 treatment sessions was given in a 12-weeks period. Manipulative therapy was applied by 8 manual therapists, who all are members of the Dutch Association of Manual Therapy (NVMT), and were considered experienced based on their previous case load.

### Clinical Assessment

Outcome measures were assessed at baseline and at 6 weeks (during the intervention period), 12 weeks (at the end of the intervention period), 26 and 52 weeks after randomization. The primary outcome measure was patient perceived recovery. Patients were considered recovered if they reported being 'completely recovered' or 'very much improved' on a 7-point ordinal scale. Secondary outcomes included the severity of main complaint [14], shoulder pain [15], functional disability [16,17] and general health [18]. Manual therapists as well as general practitioners reported their treatment on a standardized registration form.

### Economic evaluation

Cost data were collected from a societal perspective, using a cost diary that is considered valid and feasible for patient completion [19]. The diary, covering a period up to 6 weeks, was presented in a booklet form, containing instructions and an example plus a telephone number in case of questions. Patients were asked to complete the cost diary up to 26 weeks follow-up. The cost diary included direct health care costs: treatment by a general practitioner, physiotherapist, manual therapist, occupational therapist, exercise therapist (e.g. 'Mensendieck' or 'Cesar' therapist) or complementary health therapists (e.g., acupuncturist), visits to a consultant in orthopaedic surgery, neurology, rheumatology, or rehabilitation medicine, and professional home care and hospitalization. Direct non-health related costs included out of pocket expenses and costs for paid and unpaid help. Indirect costs included loss of production due to sick leave from paid and unpaid work. Indirect costs for paid work were calculated using the friction cost method [20,21], with a friction period of 123 days. Friction costs were based on the mean income and sex of the Dutch population [20,21]. We used a shadow price for unpaid work of €8.60 per hour [20,21]. A complete overview of the costs is given in the appendix (Additional file 1). Medicine costs were based on the prices of the Royal Dutch Society for Pharmacy [22].

Since participants had to visit the research centre for the clinical follow-up measurements, cost diaries were handed in at this time. A research-assistant checked the cost diaries for incompleteness and asked the patient to complete any missing data. During data check and quality control, standardized treatment registration forms completed by the general practitioner and the physical therapist were used for verification of cost data. However, when there was discrepancy between both information sources, data from cost diaries was used.

### Data analysis

Statistical analysis was performed blinded for treatment allocation, according to the intention-to-treat principle [23]. Clinical outcome measures were analyzed using a paired samples t-test (two-sided α = 0.05). Differences between groups with a 95% confidence interval were calculated for each outcome measure. Extreme values and outliers for clinical and cost data were identified and their impact on the study outcome was explored.

The cost-effectiveness analysis was performed according a protocol proposed by O'Brien and Briggs [24]. Bootstrapping was used to compare mean costs between groups and to estimate 95% confidence intervals [25,26]. Incremental cost-effectiveness ratios, that is the ratio of the difference in costs and the difference in effects between the two treatments, were determined by bootstrapping (2000 replications) according to the bias corrected percentile method [27]. The bootstrapped cost-effects pairs were graphically presented on a cost-effectiveness plane [28]. Acceptability curves were graphically reported to show the probability that a treatment is cost-effective at a specific ceiling ratio [29].

We analyzed the trial data excluding patients with missing cost data (intention-to-treat analysis). Furthermore, we conducted two adjusted analyses, once including initially excluded patients after imputation (replacement of missing cost data by the unconditional means of the treatment group) and once excluding outliers with extremely high costs.

### Role of the Funding Sources

Grant support was received from the Netherlands Organization for Scientific Research (904-65-901), and Foundation 'De Drie Lichten'. These funding organisations approved the study design, but played no part in conducting and reporting of the study.

## Results

### Patients

**Figure 1:**
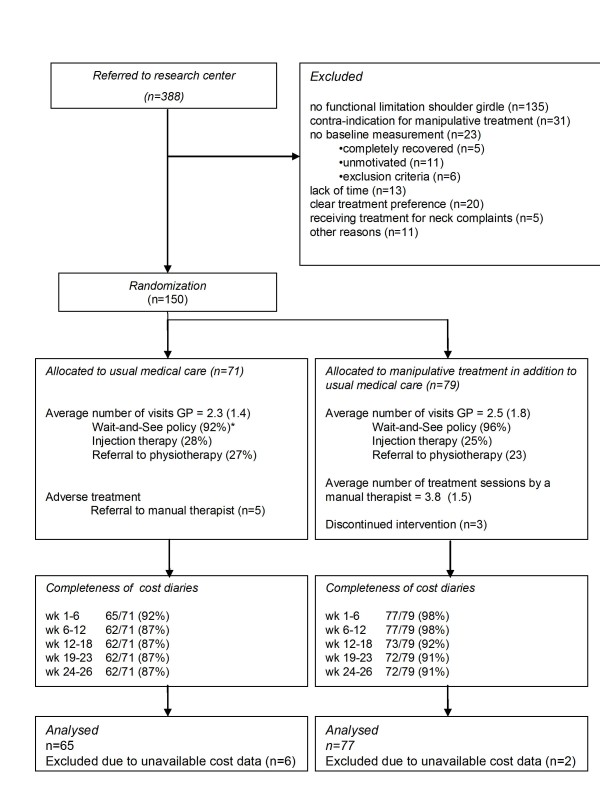
**Flow diagram of patients throughout the trial**.

A total of 150 patients were included, of which 79 patients were allocated to the AMT-group and 71 patients to the UMC-group, as shown in Figure 1. Table 1 shows their baseline data. Every patient had to complete five cost diaries that together covered 26 weeks of follow-up. In total, 645 of 750 (86%) of the cost diaries were completed and returned (See Figure 1 for details). Overall, 137 (91%) patients completed the clinical follow-up measurement at 26 weeks. A total of eight patients, six patients in the UMC-group and two patients in the AMT-group did not return any of their cost diaries and were their cost data were not imputed in the intention-to-treat analysis. These patients were generally characterized by a low mean age (42 years), a short episode of complaints (<8 weeks), a gradual onset of complaints, previous episodes of shoulder and neck complaints, and by having paid jobs (n = 5). Also they had more severe shoulder pain (mean 19 points) and rated their shoulder disability (mean 77 points) as more severe at baseline. Those patients with a similar prognostic profile but with complete cost data, reported higher total costs than patients with another prognostic profile.

**Table 1:** Baseline characteristics and values of outcome measures by allocated treatment

	*AMT (n = 79)*	*UMC (n = 71)*
Mean age in years	48.4 ± 12.4	47.8 ± 11.8
Female	42 (51)	37 (52)
Dominant side affected	58 (73)	45 (63)
Acute onset of complaints	27 (35)	19 (28)
Mean duration before consultation		
<6 weeks	28 (35)	28 (39)
6-12 weeks	25 (32)	22 (32)
12-26 weeks	10 (13)	11 (15)
>26 weeks	10 (14)	16 (20)
Previous episodes of shoulder pain		
None	18 (23)	18 (25)
1 episode	18 (23)	14 (21)
2-5 episodes	27 (34)	27 (20)
>5 episodes	16 (20)	12 (17)
Previous neck complaints	50 (63)	43 (61)
Paid job	56/79 (71%)	47/71 (66%)
Previous sick leave from work		
None	36/56 (46%)	37/47 (48%)
<2 weeks	17/56 (30%)	9/47 (19%)
>2 weeks	3/56 (5%)	4/47 (9%)
Severity of main complaint ^a^	6.9 ± 1.9	6.4 ± 2.1
Shoulder Pain ^b^	17.8 ± 4.7	17.9 ± 4.3
Shoulder Disability ^c^	58.6 ± 28.0	60.7 ± 29.0
General health (EQ5D) ^d^	0.69 ± .19	0.68 ± .18

Presented are number of patients and percentages between brackets [n (%)] or mean scores and standard deviation [mean ± SD). AMT: Manipulative therapy as add-on to UMC. UMC: Usual Medical Care by General Practitioner.

a) Severity of main complaint: rating by the patient for severity the main complaint during the preceding week (11-point scale; 0(best) -10(worst)). b) Shoulder Pain Score: rating by the patient for the experienced pain in rest, pain in motion, nightly pain, sleeping problems caused by pain, incapability of lying on the painful side, degree of radiation and general pain (all 4-point ordinal scales; 1(no pain)- 4 (severe pain)), total range: 7(best)-28 worst). c) Shoulder Disability Questionnaire: rating by the patient for the functional status of the shoulder in the preceding 24 hours (16 items; not applicable/yes/no). Presented score is the percentage of positive items in the total applicable items, total range: 0(best)-100(worst). d) EuroQol: health related quality of life (5 items; 3-point ordinal scale), total range: -1(worst)-+1(best).

### Clinical effects

In Table 2 the point estimates generally show that manipulative treatment is effective as add-on to UMC. During treatment (6 weeks) the clinical outcome measures favoured AMT in addition to UMC, but no significant differences compared to UMC alone were found. After completion of treatment (12 weeks), statistically significant more patients receiving AMT reported full recovery compared to patients receiving UMC (43% vs. 21%). At 12 weeks the difference in mean severity of main complaint and shoulder pain also favoured manipulative therapy. At 26 weeks after randomization, both groups reported similar recovery rates (41% vs. 35%), but the difference between groups in severity of main complaint (Δ = 1.2, CI: 0.2 to 2.2), shoulder pain (Δ = 0.7, CI: -1.0 to 2.5) and disability (Δ = 12.7, CI: 1.3 to 23.0) sustained.

**Table 2:** Principal outcomes at 12 weeks and 26 weeks

Outcome measure	Mean effects		Difference
	**AMT (n = 79)**	**UMC (n = 71)**	**(95%CI)**
*Perceived recovery (yes/no)*			
12 weeks	34/79 (43%)	15/71 (21%)	22% (6.9 to 35.4)
26 weeks	32/79 (41%)	25/71 (35%)	5% (-10.1 to 20.2)
*Main complaint (0 -10.)*			
12 weeks	4.4 ± 3.0	2.9 ± 3.2	1.5 (0.5 to 2.5)
26 weeks	4.7 ± 3.4	3.5 ± 3.3	1.2 (0.2 to 2.2)
*Shoulder pain (7 - 28)*			
12 weeks	5.7 ± 5.1	3.7 ± 5.2	2.0 (0.3 to 3.7)
26 weeks	5.9 ± 5.4	5.2 ± 5.5	0.7 (-1.0 to 2.5)
*Shoulder disability (0 - 100)*			
12 weeks	26.6 ± 32.3	18.2 ± 32.4	8.5 (-2.0 to 18.9)
26 weeks	33.0 ± 34.6	20.3 ± 35.9	12.7 (1.3 to 24.1)
*General Health (-1*-*+1)*			
12 weeks	0.09 ± 0.28	0.16 ± 0.25	-0.06 (-0.15 to 0.03)
26 weeks	0.11 ± 0.19	0.08 ± 0.21	0.03 (-0.04 to 0.09)

Presented are recovery rates and mean changes since baseline [± SD]). AMT: Manipulative therapy as add-on to Usual Medical Care. UMC: Usual Medical Care by General Practitioner.

### Health care consumption and sick leave

The health care consumption by the two groups is presented in Table 3. The mean number of GP visits was comparable between the two groups (1.5 visits AMT vs. 1.7 visits UMC). The mean number of unintended manual therapy in patients receiving UMC was low (0.3 visits), while the number of physiotherapy visits was higher in the AMT group (4.3 sessions) compared to the UMC group (2.0 sessions). Utilization of complementary health therapies, care from medical consultants and hospitalization were low in both groups. A difference was found in help from friends and relatives. This was mainly due to one patient allocated to AMT, who received 30 hours professional home care per week, on average for a 26 weeks period; this concerned continued care from the pre-randomisation period. Furthermore, a large difference between groups in sick leave from paid work was found: 7 patients reported sick leave in the UMC-group compared to 18 patients in the AMT-group. In the latter group, two patients reported more than 107 days of sick leave.

**Table 3:** Consumption of healthcare resources and sick leave from work during a 26-weeks follow-up period

Type of utilization	AMT (N = 77)	UMC (N = 65)
General practice [no. of visits]	1.7 ± 2.7	1.5 ± 1.4
Manual therapist [no. of visits] *	3.7 ± 2.3	0.3 ± 1.2
Physiotherapist [no. of visits]	2.0 ± 6.0	4.3 ± 8.0
Alternative therapist [no. of visits]	0.1 ± 0.7	0.3 ± 1.5
Specialist care [no. of visits]	0.2 ± 1.0	0.2 ± 4.6
Hospitalization [days]	0.0 ± 0.0	0.0 ± 0.0
Home care [hours]	0.0 ± 0.0	0.2 ± 1.4
Help from friends and relatives [hours]	7.8 ± 46.4	1.2 ± 4.6
Sick leave from paid work [days]	5.8 ± 19.2	2.7 ±10.3
Sick leave from unpaid work [hours]	13.5 ± 67.3	2.5 ± 8.6

Presented are group means and standard deviations. AMT: Manipulative therapy as add-on to UMC. UMC: Usual Medical Care by General Practitioner.

*Includes visits according the study protocol.

### Costs

Patients who were recovered at 26 weeks after randomization showed lower costs compared to patients who were not recovered (€429 vs. €1194). In patients recovered at 26 weeks follow-up the total costs were lower for patients who received AMT (€361;n = 32 vs. €508;n = 25), while in patients not recovered at 26 weeks follow-up the total costs were lower for patients who received UMC (€584;n = 47, vs. €1137;n = 47).

The mean and standard deviations as well as mean differences and their confidence intervals of the costs for each intervention group are presented in Table 4. Treatment by general practitioner and physiotherapist accounted for 21% of the total costs in the UMC group and for 6% in the AMT group. In addition the protocolised manipulative therapy also represents 6% of the total costs in the latter group. The total direct costs cost were higher in patients who received AMT, but these differences were not statistically significant. A large proportion of the total costs are due to indirect costs (69% for UMC-group vs. 78% for AMT-group). The large difference in indirect costs (€523) was for a considerable part due to two patients allocated to AMT. These were outliers with an average total cost of €18,408 per patient.

**Table 4:** Mean costs (in €) per treatment group and mean differences during a 26 week follow-up period

Costs	AMT	UMC	AMT vs. UMC
	**Mean ± SD**		**Mean difference (95%CI)**
**Total costs**			
Intention-to-treat (n = 142)	1167 ± 3348	555 ± 1290	612 (-193 to 1581)
Adjusted for outliers (n = 140)	676 ± 1445	555 ± 1290	121 (-340 to 581)
**Total direct costs**			
Intention-to-treat (n = 142)	293 ± 602	192 ± 232	101 (-75 to 252)
Adjusted for outliers (n = 140)	266 ±555	192 ± 232	74 (-72 to 221)
**Direct health care costs**			
Intention-to-treat (n = 142)	174 ± 190	153 ± 185	21 (-28 to 48)
Adjusted for outliers (n = 140)	166 ±180	153 ± 185	14 (-47 to 75)
**Direct non-health care costs**			
Intention-to-treat (n = 142)	119 ± 468	39 ±119	80 (-25 to 191)
Adjusted for outliers (n = 140)	99 ± 436	39 ± 119	60 (-49 to 170)
**Indirect costs**			
Intention-to-treat (n = 142)	913 ± 3091	381 ± 1222	523 (-338 to 1329)
Adjusted for outliers (n = 140)	446 ± 1138	381 ± 1222	66 (-329 to 460)

€: Euro's (€1 = 1.20 US Dollars); AMT: Manipulative therapy as add-on to UMC. UMC: Usual Medical Care by General Practitioner.

### Cost-effectiveness analyses

The cost-effectiveness ratios (CER) for the comparison between AMT and UMC are presented in Table 5. The CER for perceived recovery suggests that the incremental costs are €19,773 per patient to recover with AMT after 26 weeks. The CER for shoulder disability shows the lowest incremental costs for manipulative therapy (€47 per unit improvement).

**Table 5:** Cost-effectiveness ratios for clinical outcome measures

Outcome measure	AMT vs. UMC	
	Intention-to-treat	Adjusted for outliers
Perceived recovery	19773	2876
Main complaint	1261	215
Shoulder pain	934	175
Shoulder disability	47	5
General health	16002	2952

AMT: Manipulative therapy as add-on to UMC. UMC: Usual Medical Care by General Practitioner.

**Figure 2:**
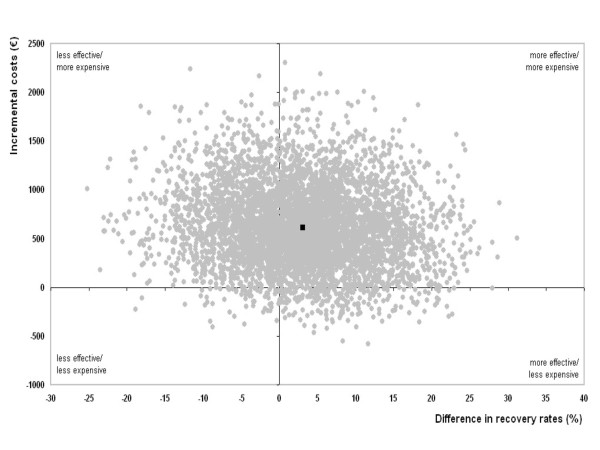
**Cost-effectiveness plane for patient perceived recovery for manipulative therapy in addition to usual medical care by the GP compared to usual medical care alone**.

Figure 2 displays the cost-effectiveness plane for patient perceived recovery. It represents 5000 replications of the CER for perceived recovery comparing the two treatment modalities. Most cost-effect pairs are concentrated around zero on the Y-axis, suggesting that there is only a minor clinical effect, and above zero on the X-axis, suggesting that treatment with AMT generates more costs than UMC alone. The other outcome measures demonstrated a similar graph. However, as shown in Table 3, the clinical effects for severity of main complaint, shoulder pain and shoulder disability are more prominent at 26 weeks after AMT than UMC.

**Figure 3:**
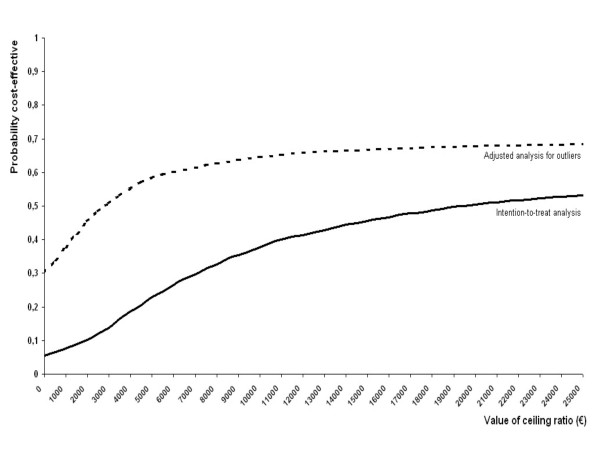
**Cost-effectiveness acceptability curves for patient perceived recovery**.

The cost-effectiveness acceptability curves for patient perceived recovery (Figure 3) show the probability that manipulative therapy in addition to UMC is cost-effective at a certain cost ceiling ratio. For example, in the unadjusted analysis the probability that AMT is cost-effective is approximately 35% at a ceiling ratio of €10,000. For patient perceived recovery, the 50%-probability corresponds with a ceiling ratio of €20,000. For the other outcome measures, the 50%-probability of cost-effectiveness is: €1150 for severity of main complaint, €850 for severity of shoulder pain, €45 for severity of shoulder disability and €16,030 for general health.

### Adjusted analyses

Besides the intention-to-treat analysis, two adjusted analyses were performed. In the first adjusted analysis the missing cost data and clinical data of the eight excluded patients were imputed. This analysis showed no important differences from the analysis (no data presented). In the second adjusted analysis, the two patients allocated to AMT who generated extremely high costs due to sick leave from work were excluded. Mainly due to lower indirect costs this analysis showed a decrease of the difference in total costs between the intervention groups from €612 to €121, whereby the cost-effectiveness ratio for perceived recovery decreased from €19,773 to €2,876, and the value of the ceiling ratio of 50%-probability that AMT is cost effective to recover one patient after 26 weeks decreased from €20,000 to €2,800. Vice versa, a ceiling ratio of €10,000 corresponds with a probability of successful recovery of 35% in the main analysis compared to 65% in the sensitivity analysis.

## Discussion

Manipulative therapy of the cervicothoracic spine and the adjacent ribs as add-on to usual medical care by the general practitioner accelerates recovery of shoulder complaints. The differences between AMT and UMC were largest for recovery after 12 weeks (22%) and severity of main complaint and shoulder disability after 26 weeks (12% and 13%, respectively). However, AMT was associated with higher costs than UMC. The balance between costs and effects is markedly influenced by very few extreme values for costs and few missing data. Adjustment for these extremes cost outliers markedly changed the balance between costs and effects: rather favourable results (lower total costs) for AMT were obtained. Given the large influence of these extreme values associated with non-shoulder morbidity, we argue that the adjusted analysis is justified and represent the cost-effectiveness for AMT for shoulder complaints more accurately.

The higher costs of the manipulative therapy group cannot completely be explained by the costs for the AMT alone. The manipulative therapy (mean = 3.7 sessions) accounts for €270, which explains 43% of the difference in total costs between both groups. Although these differences were not statistically significant, the AMT group generated higher costs on total health related care as well as higher indirect costs than the UMC-group. As expected, the direct health related care costs were the highest during the intervention period.

The UMC treatment represents 21% of the total costs for patients that received UMC only and 6% for patients that received manipulative therapy in addition to UMC. In the manipulative therapy group, only 6% of the total costs were due to the standardized manipulative therapy. The largest proportion of the total cost was due to sick leave from work: 61% in patients that received UMC only and 65% in patients that were allocated to manipulative therapy.

Sample size calculations were not based on demonstrating cost-effectiveness. We needed 250 patients (125 in each treatment group) for sufficient statistical power [30] to detect a clinically relevant difference of 20% between groups, taken into account a recovery rate of 50% in the UMC group after 26 weeks, a two-sided alpha of 0.05, a statistical power (1-β) of 0.80 and an attrition rate of 10%. An appropriately powered cost-effectiveness study could require a much larger sample size [31]. Although, because of constraints of time and funding we included 150 patients with high adherence to the allocated treatment and very limited attrition during follow-up, clinically relevant effects have been found. Moreover, due to adequate response and crosschecking of collected data we compiled a rather complete data set. We were unable to collect any of the cost diaries of 8 patients, of whom 6 received UMC alone and 2 received AMT. These 8 patients had a prognostic status that is associated higher total costs. After imputation of missing data and adjustment for extreme values, a rather small difference in total costs between the groups remained. Therefore, extreme and missing cost data resulted in an underestimation of the cost-effectiveness of manipulative treatment as an add-on to UMC.

This economic evaluation was based on the clinical outcome at 26 weeks after randomization and therefore did take into account the short-term improvement. Since manipulative therapy aims to restore the function of concurrent dysfunctions of the neck and high back, which is an important prognostic indicator for shoulder disorders [32,33], we expected manipulative therapy to be cost saving on the long term. Perhaps with a longer follow-up more cost savings can be obtained. But our follow-up for costs was limited to 6 months due to time constraints. It is possible that prolonged and recurrent pain episodes generate additional costs for more expensive care, e.g. diagnostic imaging and surgical treatment, including hospitalization.

The present study is the first to evaluate the cost-effectiveness of a manipulative treatment in shoulder complaints alongside a randomized trail. Korthals and colleagues evaluated the cost effectiveness of physiotherapy, manual therapy, and care by a general practitioner for patients with neck pain in a randomized trial [34]. They concluded that manual therapy, (notably spinal mobilization) was more effective and less costly for treating neck pain than physiotherapy or care by a general practitioner.

## Conclusion

Our study shows that manipulative therapy in addition to UMC accelerates recovery, is slightly more effective on the long term, but is associated with higher costs compared to UMC alone up to 26 weeks after initiation of treatment. The balance between costs and effects was markedly influenced by very few extreme values. After adjustment for these extreme values the differences in costs were small and, consequently, the extra costs for one additional recovery relatively low.

## Competing interest statement

The authors declare that they have no competing interests.

## Authors' contributions

Study concept and design: GJDB, JCW, BMJ, KP, GJMGH; Collection and assembly of data: GJDB; Provision of study materials or patients: JCW; Analysis and interpretation of the data: GJDB, MWT, GJMGH; Drafting of the article: GJDB; Critical revision of the article for important intellectual content: GJDB JCW MWT BMJ KP GJMGH; Final approval of the article: GJDB JCW MWT BMJ KP GJMGH; Statistical expertise: MWT, GJMGH; Obtaining of funding and Study supervision: JCW, GJMGH; GJMGH had full access to all of the data in the study and takes responsibility for the integrity of the data and the accuracy of the data analysis.

## Pre-publication history

The pre-publication history for this paper can be accessed here:

http://www.biomedcentral.com/1471-2474/11/200/prepub

## Supplementary Material

Additional file 1 **Appendix 1 Cost used in the economic evaluation**. €: Euro's; FCM: friction cost method Click here for file
