# Supplementary material for: Manipulative therapy in addition to usual medical care accelerates recovery of shoulder complaints at higher costs: economic outcomes of a randomized trial
Source: BMC Musculoskelet Disord. 2010 Sep 6;11:200. doi: 10.1186/1471-2474-11-200 (PMC2944217; doi:10.1186/1471-2474-11-200)
Supplement: Additional file 1 — Appendix 1 Cost used in the economic evaluation. €: Euro's; FCM: friction cost method [file 1471-2474-11-200-S1.DOC]

| **Costs** | **€** |
| --- | --- |
| *Direct health care costs* |  |
| General Practitioner (max 10 min) | 18.97 |
| Manual therapist (max 20 min) | 19.65 |
| Other therapists (max 30 min)  (physiotherapist, ‘Mensendieck’, ‘Cesar’ and occupational therapist) | 19.65 |
| Specialists  (orthopaedist, neurologist, rheumatologist, and physician for rehabilitation medicine) | 44.21 |
| Hospitalization (per day) | 236.22 |
| *Direct non-health related costs* |  |
| Alternative therapists (acupuncturist, homeopath, chiropractor, and others) | As indicated by patient |
| Home care | 8.60 |
| Help from partner/relatives/friends | 8.60 |
| *Indirect costs* |  |
| Sick leave from paid job | FCM |
| Sick leave from unpaid work | 8.60 |
